# Susceptibility to malaria during the prevention of re-establishment phase in Sri Lanka

**DOI:** 10.1186/s12936-022-04127-4

**Published:** 2022-03-27

**Authors:** Hamsananthy Jeevatharan, Rajitha Wickremasinghe

**Affiliations:** 1 Ministry of Health, Suwasiripaya, 385 Rev. Baddegama Wimalawansa Thero Mawatha, Colombo, 01000 Sri Lanka; 2 Department of Public Health, Faculty of Medicine, University of Kelaniya, Thalagolla Road, P.O. Box 6, Ragama, 11010 Sri Lanka

**Keywords:** Malaria parasite, Social vulnerability, Susceptibility, Prevention of re-establishment of malaria, Sri Lanka

## Abstract

**Background:**

Sri Lanka eliminated malaria in November 2012 and was certified malaria-free by the World Health Organization (WHO) in September 2016 but is facing a challenge to prevent re-establishment of malaria. Influx of travellers from malarious countries and the presence of malaria vectors in formerly endemic areas make the country both receptive and vulnerable. Susceptibility to malaria, the predisposition of populations to be infected by malaria parasites, is influenced by biologic and generic factors such as the age-sex composition, socio economic status, and the migration history of the population. The aim of this study was to assess susceptibility to malaria during the prevention of re-establishment phase in Sri Lanka.

**Methods:**

A national survey was conducted among 3454 households. A multistage cluster sampling technique was used to select the households. Susceptibility was assessed based on pre-defined variables by interviewing heads of households using an interviewer-administered questionnaire. Basic socio-demographic information, travel history, history of fever and past malaria infections in the preceding three years were collected. Data were analysed using SPSS version 20 package.

**Results:**

The percentage of the population who had been overseas within the last 3 years in the urban sector (4.5%, n = 99) was higher than that of the rural (2.8%, n = 288) and estate sectors (0.2%, n = 2) (p < 0.001); it also declined with the wealth index up to the 4th quintile with a slight rise in the 5th quintile (p < 0.001). The likelihood of travel overseas was 1.75 times (95% CI: 1.38–2.22) higher for urban residents as compared rural estate residents; it was 1.46 times (95% CI: 1.16–1.92) higher for persons from the upper wealth index quintile as compared to persons from the 1st and 2nd quintiles after controlling for sex, age and area of residence. 177 persons had fever within the past 2 weeks of the survey. There was no association between presence of fever within the last 2 weeks and sector or travel abroad.

**Conclusions:**

Urban residents, upper socioeconomic class persons and males are more likely to travel overseas and bring the parasite into the country. Social vulnerability and risk of re-establishment of malaria can be assessed by combining susceptibility with resilience and receptivity.

## Background

The burden of malaria has decreased in many countries over the last decade due to accelerated prevention and control activities. In 2019, there were about 229 million cases of malaria and an estimated 409,000 deaths due to malaria [1]. Over the last decade, 10 countries have been certified by the World Health Organization (WHO) as malaria-free: Morocco (2010), Turkmenistan (2010), Armenia (2011), Maldives (2015), Sri Lanka (2016), Kyrgyzstan (2016), Paraguay (2018), Uzbekistan (2018), Argentina (2018) and Algeria (2019) [1]. However, there has been no significant progress in reducing the global malaria burden during the period 2015–2017 [2]. The achievement of malaria elimination carries with it the risk of re-establishment of malaria. The risk of re-establishment of malaria is a challenge for countries that have eliminated malaria until global eradication of malaria is achieved.

Sri Lanka was certified as malaria-free in September 2016 with no indigenous case of malaria being reported since November 2012. An indigenous case is a locally contracted case with no evidence of importation and no direct link to transmission from an imported case. An imported case is a malaria case or infection in which the infection was acquired outside the area in which it is diagnosed [2, 3]. Less than 100 imported malaria cases have been reported annually from 2013 onwards, among both Sri Lankans travelling overseas and foreign nationals coming into the country, maintaining the susceptibility of the country to malaria in a receptive environment [4–6]. There was one introduced malaria case (a locally contracted case with strong epidemiological evidence linking it directly to a known imported case i.e., first-generation local transmission) in December 2018 [7]. Thus, there is a potential threat for the re-establishment of malaria in Sri Lanka.

The understanding of the malariogenic potential of a country is essential for implementation of cost-effective interventions to prevent re-establishment of malaria. The malariogenic potential is the degree of risk of malaria transmission which depends on receptivity, vulnerability and infectivity. Receptivity to malaria transmission is determined by the presence of competent vectors, a suitable climate and a susceptible population. Vulnerability refers to the rate of importation of parasites through the movement of infected individuals or, occasionally, infected Anopheline vectors. Infectivity, or vector susceptibility, depends on the compatibility between the Anopheline vector and the infecting *Plasmodium* strain [3, 8]. A summary of the definitions used is given in Box 1.

**Table:** 

Box: 1 • Malariogenic potential is the level of transmission in a given area arising from the combination of malaria receptivity, vulnerability and infectivity [3] • Receptivity of an ecosystem to transmission of malaria–having the presence of competent vectors, a suitable climate and a susceptible population [3] • Vulnerability is the frequency of influx of infected individuals or groups and/or infective anopheline mosquitoes [3] • Infectivity or vector susceptibility is the compatibility between the Anopheles vector and the infecting strain of Plasmodium [3] • Social Vulnerability is the characteristics of a person or group (susceptibility) and their capacity to anticipate, cope with, resist and recover (resilience) from the impact of a natural hazard [21] • Susceptibility is the predisposition of the population being negatively affected by an outbreak, includes biologic and generic factors [9]	

### Susceptibility to malaria

Susceptibility is determined by biological susceptibility factors such as age and sex, and generic susceptibility factors such as migration and socio economic status of the population [9].

**Fig. 1:**
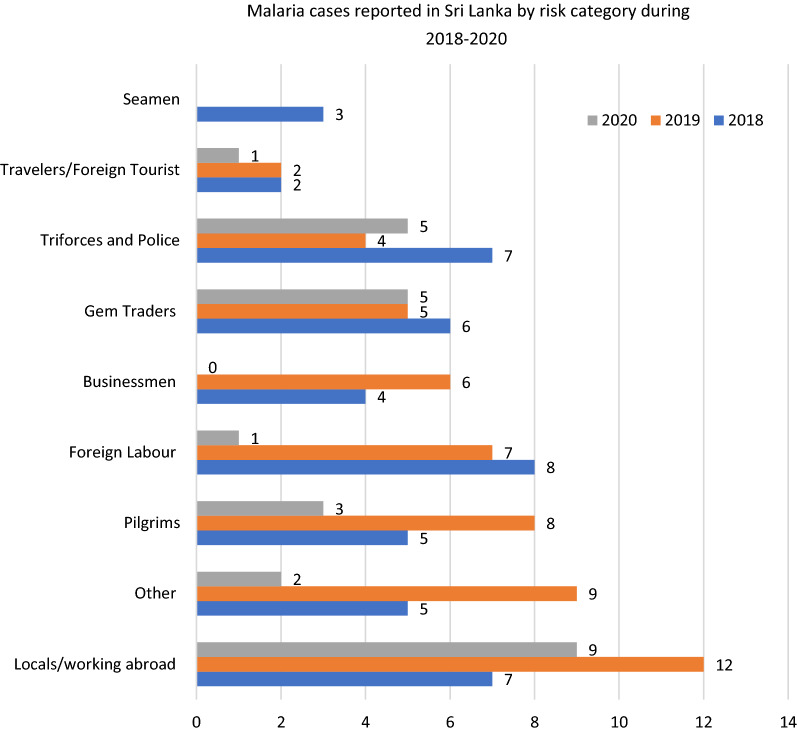
Malaria cases reported in Sri Lanka by risk category during 2018–2020 [11]

Migration of people between Sri Lanka and malaria endemic countries is the most important susceptibility factor in the prevention of re-establishment of malaria phase. It is the main route of entry of malaria parasites into the country after elimination. The risk groups in whom imported malaria was reported include; asylum-seekers, local fishermen who returned from Sierra Leone, army personnel returning after serving in UN peace-keeping missions in Haiti and South Sudan, irregular migrants from Myanmar, foreign skilled and unskilled labour working in several parts of the country, Sri Lankan nationals engaged in business in African countries and in India, tourists and foreign labour [10]. Figure 1 shows the reported malaria cases by risk category during 2018–2020 [11].

The importance of social factors that make some groups or individuals more susceptible to infection and more limited in their ability to respond to illness than others was highlighted in a study of social vulnerability assessment of vector-borne diseases [9]. In Sri Lanka, being an island nation, malaria is no more a ‘disease of the poor’ because of the influence of social class on migration. The number of travellers from malaria endemic countries has a direct impact on vulnerability to malaria in a receptive area. Children, pregnant women and immuno-compromised persons, such as HIV/AIDS and tuberculosis patients are the more susceptible groups, in general [12].

Fernando quotes that "only in retrospect has it become fully clear that the failure of malaria eradication was in large part a failure at the social and organization levels” [13]. Several studies have shown that socio-cultural factors should have been taken into account at the initial stage of the programme and for the recommendation of programme design [14].

There are no published studies on susceptibility to malaria during the prevention of re-establishment phase in Sri Lanka. There are some studies which assessed the influence of certain social aspects on malaria control during the control phase [15, 16]. This study assessed the biological and generic (relating to or characteristic of a whole group or class) susceptibility to malaria during the prevention of re-establishment phase in Sri Lanka.

## Methods

### Study setting

Sri Lanka is an island nation in the Indian Ocean, southeast of India, with a total land area of 65,610 km^2^. The population of Sri Lanka is approximately 21 million. Approximately 18% of the population is resident in urban areas [17]. Administratively, Sri Lanka is divided into 9 provinces, and the 9 provinces are further divided into 25 districts. The Medical Officer of Health (MOH) is responsible for preventive health services in a defined area. The MOH area is further divided into Public Health Inspector and Public Health Midwife (PHM) areas [4].

This cross-sectional study covered the whole country. The study population consisted of members of all households in all districts of Sri Lanka. A household was defined as ‘one or more persons living together and who have a common arrangement for provision of food living in a housing unit’ [18]. Public places, such as homes for elderly, orphanages, and religious homes, were excluded.

### Conceptual setting: susceptibility as a domain of the social vulnerability framework

Social vulnerability is a developing concept on communication of natural hazards and disasters and is partially the result of social inequalities. Social factors influence susceptibility of various groups and their ability to respond to harm [19]. Social vulnerability is not only due to exposure to hazards alone, but also depends on the sensitivity and resilience of the system to prepare, cope and recover from such hazards [20]. Unlike the WHO definition of vulnerability related to malaria, Wisner et al. define social vulnerability as “the characteristics of a person or group in terms of their capacity to anticipate, cope with, resist and recover from the impact of a natural hazard” [21]. If the influx of infected persons and mosquitoes in a malaria eliminated region are considered as a natural disaster, then social vulnerability is a measure of how communities anticipate, cope with and recover from an outbreak if there is one. Methods for the Improvement of Vulnerability Assessment in Europe (MOVE) project has elaborated a framework in the context of natural hazards and climate change [9].

**Fig. 2:**
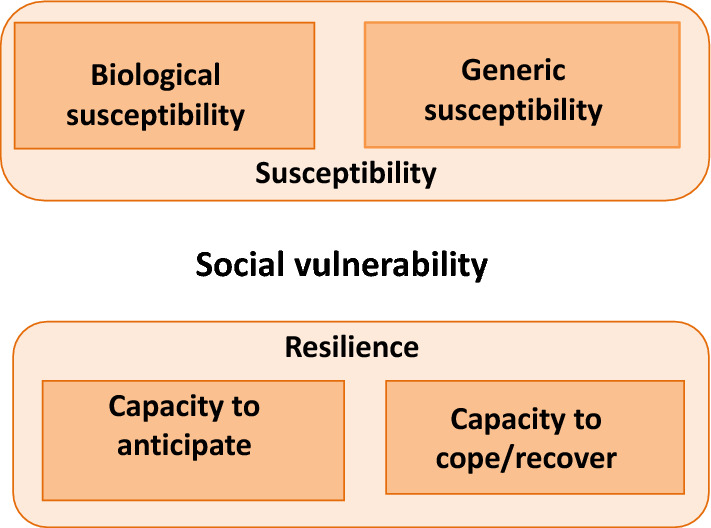
Conceptual framework for social vulnerability adapted from Kienberger and Hagenlocher [22]

Susceptibility domains were adapted from the conceptual framework for social vulnerability to vector borne diseases by Kienberger & Hagenlocher (Fig. 2) [22], who characterize social vulnerability in two interrelated domains, and biological susceptibility, and lack of resilience as the predisposition of the population [10, 23]. While the biological susceptibility factors predispose a community to the malaria burden, the resilience factors determine the community’s ability to anticipate, respond to, cope with, or recover from the malaria burden.

### Adapted framework for susceptibility to malaria during the prevention of re-establishment phase`

**Fig. 3:**
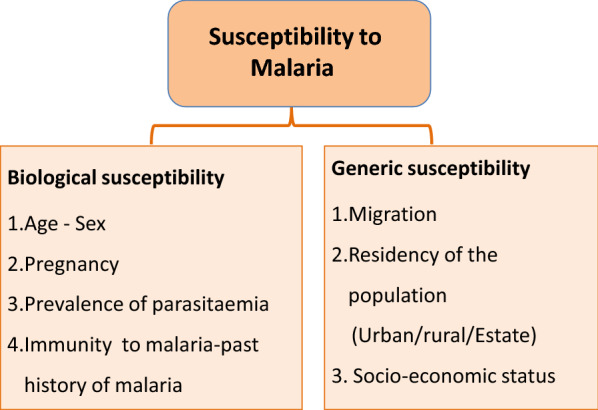
Adapted framework for susceptibility to malaria during the prevention of re-establishment phase from Kienberger and Hagenlocher [22]

Age, gender, pregnancy status, prevalence of parasitaemia and history of malaria (immunity status) during the past 3 years were considered as biological susceptibility indicators. Migration, socio-economic status and residency of the population (urban/rural/estate) were considered as generic susceptibility indicators (Fig. 3). Urban areas were defined as all areas administered by municipal and urban councils. ‘Pradeshiya Sabhas’ were included under the rural sector and the plantation areas were considered as the estate sector [18].

### Development of the data collection tool

Based on the conceptual framework, a household survey questionnaire was developed to assess susceptibility to malaria during the prevention of re-establishment phase. The following details were collected: identification of households including socio demographic characteristics of household members, and household characteristics to assess socio-economic status (SES). The assessment of socio economic status (SES) was based on the Demographic Health Survey (DHS) format used worldwide [24]. Wealth index was used to classify the socio-economic status of participants. The source of water, toilet facilities, type of fuel used, material used for the floor of the house, mode of transport, access to mass media, electricity in the household, possession of some household items, and ownership of agricultural land and farm animals were included in the questionnaire. The migration history of household members within the last 3 years and history of fever prior to two weeks of survey was included.

### Validation of tool

Questionnaires along with the adapted conceptual framework were distributed among malaria experts to assess judgmental validity (face and content) of the questionnaire. The questionnaires were translated using standard methods and pre-tested in the field; necessary changes were made accordingly.

### Sample size and sampling

For sample size calculation to assess susceptibility to malaria, there are no guidelines or literature that could be used in the prevention of re-establishment phase. Hence, the number of Sri Lankans travelling overseas was considered the most important variable for sample size calculation. In 2014, 1,311,258 Sri Lankans departed from the Bandaranaike International Airport, which was about 6% of the estimated population [25]. Therefore, to estimate the proportion of Sri Lankans travelling overseas in a year as 6%, with a margin of error ranging from 4.5 to 7.5% for a 95% confidence interval, assuming a design effect of 3.2 for using cluster sampling (cluster size of 12), and a non-response rate of 10%, a sample of 3424 households had to be surveyed [26]. Households were selected equally from among the 25 districts proportionate to population in urban, rural and estate sectors. Therefore, from each district 137 households had to be surveyed from 11.4 clusters per district. Hence, twelve clusters of 12 households from each cluster were randomly selected from each district (25 districts × 12 PHM clusters × 12 households) to give a total sample size of 3600 households.

A stratified multistage cluster sampling method was used with the primary sampling unit being MOH areas. 6–8 MOH areas were randomly selected from each district. 12 PHM area clusters were randomly selected from the selected MOH areas (on average 2 PHM clusters per MOH area). From each PHM area cluster, the starting point of the household survey was randomly selected by dropping a headed pin on the PHM area map and the house closest to the pointed edge was selected. After the first house was identified, every tenth house to the left of the selected house was chosen until 12 households for that PHM area were surveyed.

### Data collection

The validated tool was used in the household survey in all districts of the country. The data collectors were trained on sampling technique and the importance of sampling during the training sessions; in the field they were supervised by malaria officers or the principal investigator. An interviewer manual and a Global Positioning System (GPS) data collection format were prepared based on the guidelines for conducting Malaria Indicator Surveys as given by the Roll Back Malaria Initiative and given to all data collectors for easy reference. Fieldwork was carried out from July 2016 to March 2017.

The PHM of the area informed the heads of selected households about the survey and the date of interview. On the assigned date, data collectors visited the households and interviewed the head of the household. If the head of the household could not be interviewed (e.g. household closed), the next closest household was selected. If the head of the household had responded and was not available at the time of survey, the contact number was obtained; the household head was approached a second time, after confirmation of his/her presence, mainly during weekends. If the second attempt failed, the house was visited for a third time; if the head of the household could not be contacted a third time, it was considered a non-responder.

Screening for malaria was done according to the standard operating procedures for parasitological screening for malaria of the Anti Malaria Campaign (AMC), Sri Lanka. Blood films were taken from all the household members who were present at the time of visit. Both microscopy and RDTs were used for screening. RDTs were used in areas that did not have microscopy facilities. Microscopy was done by a trained Public Health Laboratory Technologist (PHLT) from each region. Data collection was supervised by regional malaria officers, medical officers attached to Anti Malaria Campaign/Headquarters and the principal investigator.

### Data analysis

Data analysis was done using IBM SPSS statistics version 20 software package. Descriptive analyses were used to describe socio-demographic characteristics. Socio-economic status of the population was assessed by the wealth index using exploratory factor analysis. Principal components analysis (PCA) was used to generate a weighted score based on household assets. The wealth index for a household is the linear combination defined as the principal component variable across households or individuals with a mean of zero and a variance of one, corresponding to the ‘Eigenvalue’ of the correlation matrix. The estimated wealth index was based on a population of 13,365 resident in 3,454 households located in all districts in Sri Lanka.

The variables selected for derivation of the wealth index were based on the methodology used by the Department of Census and Statistics in their routine surveys (DHS) [17] and in the guidelines to conduct a Malaria Indicator Survey [27].

Descriptive analyses were used to describe variables included in ascertaining biological and generic susceptibility. Weighted proportions of susceptibility indices were calculated based on sector of residency, considering the number of household members in each sector.

The association between wealth index, sector of residency and history of migration was analysed using the chi-square test. A multivariate analysis was done using a binary logistic regression model with travel abroad as the dependent variable to identify factors associated with overseas travel using age (categorized into 3 groups as < 5 years and > 65 years, 6–35 years and 35–65 years), wealth index (categorized into three groups as wealth quintiles 1 and 2, wealth quintiles 3 and 4 and wealth quintile 5) and sector of residency.

## Results

**Fig. 4:**
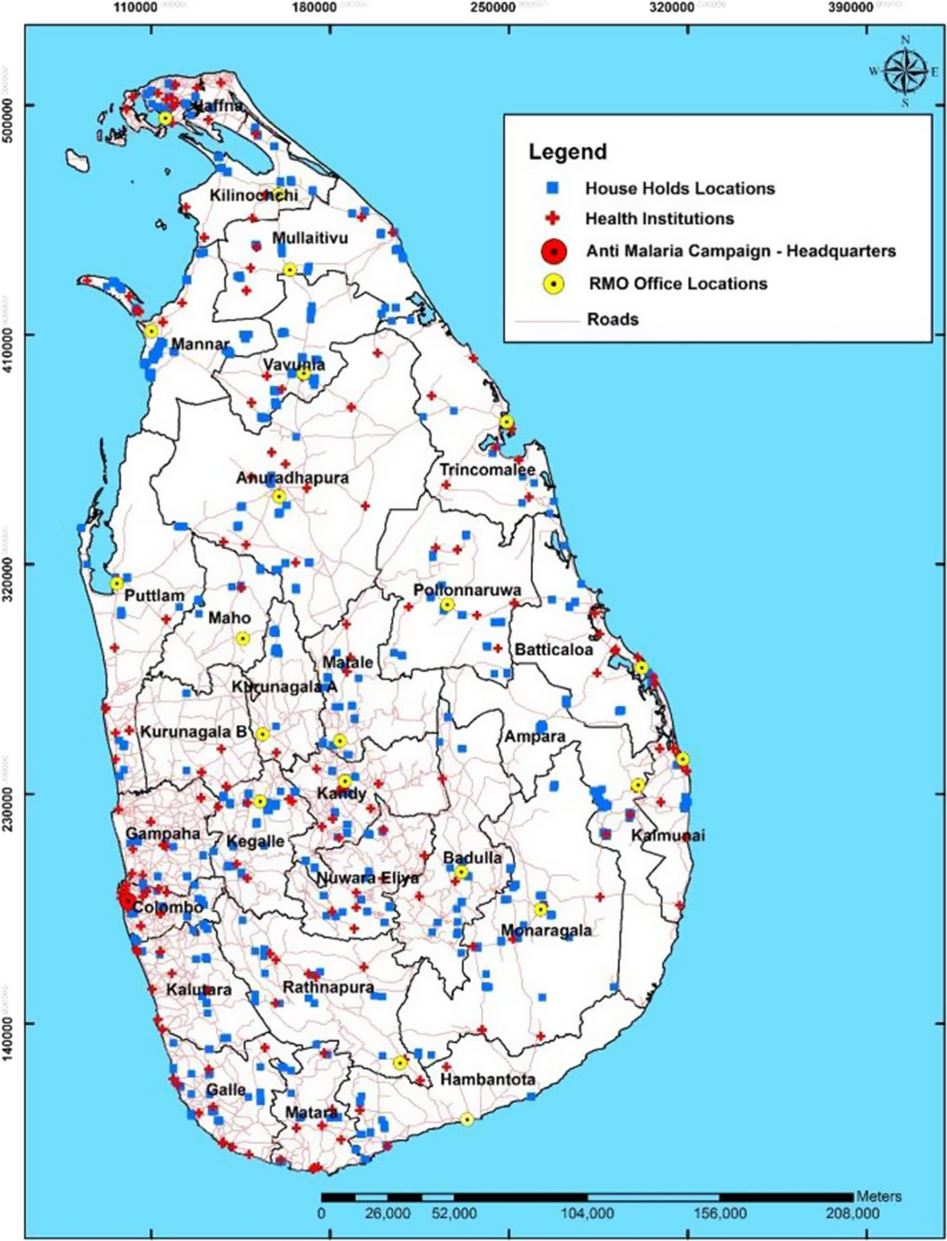
Geographic distribution of surveyed households

3,454 out of the selected 3,600 households (response rate of 95.9%) responded within two consecutive visits. Figure 4 shows the geographic distribution of the surveyed households.

### Characteristics of the population of the household survey

The mean age of the heads of households was 51 years (SD = 13.9), the ages ranging from 19 to 92 years. The majority of the heads of households were males (n = 2758; 79.8%); most heads of households were 41–60 years of age (n = 1317, 38.1%) (Table 1). The age-sex distribution of the surveyed population was similar to the national population estimates for 2017 (sample < 5 years–8.7% vs 8.6% national estimate; sample > 65 years–7.7% vs 7.9% national estimate) [28].

**Table 1:** Distribution of heads of households and household members by age and sex

Age Group	Number of Males (%, 95% CI)	Number of Females (%,95% CI)	Total Number (%,95% CI)
*Heads of Households*			
< 20	6 (0.2, 0.1–0.4)	0 (0.0)	6 (0.2,0.13–0.23)
21–40	759 (22.0,20.6–23.4)	135 (3.9, 3.3–4.6)	894 (25.9,24.4–27.4)
41–60	1317 (38.1,36.5–39.8)	317 (9.2,8.2–10.2)	1634 (47.3,45.6–49.0)
> 60	676 (19.6,18.3–20.9)	244 (7.1,6.2–8.0)	920 (26.6,25.2–28.1)
Total	2758 (79.8, 78.5–81.2)	696 (20.2,18.8–21.5)	3454 (100)
*Household Members*			
< 5	627 (4.7,4.3–5.1)	536 (4.0,3.7–4.4)	1163 (8.7,8.2–9.2)
6–20	1556 (11.6,11.1–12.2)	1455 (10.9,10.4–11.4)	3011 (22.5,21.8–23.3,)
21–35	1529 (11.4,10.9–12.0)	1684 (12.6,12.0–13.2)	3213 (24.0,23.3–24.8)
36–50	1311 (9.8,9.3–10.3)	1389 (10.4,9.9–10.9)	2700 (20.2,19.5–20.9)
51–65	1098 (8.2,7.8–8.7)	1157 (8.7,8.2–9.2)	2255 (16.9,16.2–17.5
> 65	519 (3.9,3.6–4.2)	504 (3.8,3.5–4.1)	1023 (7.7,7.2–8.1)
Total	6640 (49.7,48.8–50.5)	6725 (50.3,49.5–51.2)	13,365 (100)

The majority of heads of households was Sinhalese (n = 2,240, 64.9%), married (n = 3032, 87.8%) and employed (n = 2,995, 86.8%) (Table 2). Seventy eight percent (n = 2693) of heads of households had more than primary education while 3.9% (n = 133) had no schooling. The majority of families (n = 2,223, 64.4%) had a monthly income between Sri Lankan Rupees (SLR) 10,000–50,000 (Table 2). The majority of households (n = 2,685, 77.7%) and the household members (n = 10,231, 76.5%) were from the rural sector (Table 3).

**Table 2:** Socio-demographic characteristics of heads of households

Characteristic		Number (%,95% CI) (n = 3454)
Ethnicity	Sinhalese	2240 (64.9, 63.2–66.5)
	Tamil	1001 (28.9, 27.5–30.5)
	Muslim	210 (6.1, 5.3–6.9)
	Other	3 (0.1,0.02–0.25)
Marital status	Married	3032 (87.8, 86.6–88.7)
	Unmarried	62 (1.8, 1.4–2.3)
	Divorced/Separated	57 (1.7, 1.3–2.1)
	Widowed	303 (8.8, 7.9–9.8)
Educational level	No schooling	133 (3.9, 7.9–9.8)
	Primary (Grade1–5)	628 (18.2, 16.9–19.5)
	Secondary(Grade 6–10)	1019 (29.5, 28.0–31.1)
	Completed GCE^1^O/L^2^or equivalent	1060 (30.7, 29.3–32.3)
	Completed GCE A/L^3^or equivalent	532 (15.4, 14.2–16.6)
	Degree and above	82 (2.4, 1.9–2.9)
Occupation	Employed	2995 (86.8, 85.5–87.8)
	Retired	187 (5.4, 4.7–6.2)
	Unemployed	272 (7.8, 7.0–8.8)
Monthly Family Income (SLR)^4^	< Rs 10,000/-	945 (27.4,25.9–28.9)
	Rs 10,001/-–Rs 30,000/-	1505 (43.6, 41.9–45.3)
	Rs 30,001/-–Rs 50,000/-	718 (20.8, 19.4–22.2)
	Rs 50,001/-–Rs 70,000/-	212 (6.1, 5.4–7.0)
	Rs 70,001/-–Rs 100,000/-	64 (1.9, 1.4—2.4)
	> Rs 100,000/-	10 (0.3, 0.1–0.5)

^1^General Certificate of Education, ^2^Ordinary Level,^3^Advanced level

^4^ SLR refers to Sri Lankan rupees (1 USD ≈ SLR 150 at time of study)

**Table 3:** Distribution of households by urban, rural and estate sectors

Sector	Number of households (%,95% CI)	Number of household members (%,95% CI)
Urban	573 (16.7, 15.4–17.8)	2201 (16.5, 15.8–17.1)
Rural	2685 (77.7, 76.3–79.1)	10,231 (76.5, 75.8–77.3)
Estate	196 (5.7, 4.9–6.5)	933 (7.0, 6.6–7.4)
Total	3454 (100.0)	13,365 (100.0)

### Susceptibility indices

Susceptibility indices were calculated as weighted proportions, weightings based on sector of residency (Table 4).

**Table 4:** Weighted susceptibility indices

Susceptibility indicators	% (95% CI) (N = 13,365)
*Biological Susceptibility Indicators*	
Age under 5 and over 65	16.35 (15.63–17.09)
Male gender	49.67 (48.69–50.65)
Women of childbearing Age	26.50 (25.64–27.38)
Pregnant mothers	36.0* (35.06–36.95)
*Parasitaemia status*	
Microscopy done (n = 12,420)	93.2 (92.69–93.69)
Microscopy positive cases among tested	0 (0.0)
RDT** done (n = 6069)	53.83 (52.85–54.81)
RDT** positive among tested	0 (0.0)
Prevalence of fever 2 weeks prior to the survey (n = 177)	1.37 (1.15–1.62)
Microscopy positive cases among fever patients	0 (0.0)
Past history of malaria within 3 years	0 (0.0)
*Generic Susceptibility Indicators*	
Been abroad within last 3 years (n = 386)	3.01 (2.68–3.36)
Upper wealth Quintiles (5)	20.32 (19.54–21.12)

^*^Pregnancy per 1000 women of child bearing age (15–49 years)

^**^Rapid Diagnostic Test

#### Biological susceptibility indices

There were 177 persons (1.35%, 95% CI 1.16–1.56) who had history of fever within the last 2 weeks, and no one gave a past history of malaria within the last 3 years of the survey. There were no malaria cases detected among the survey population or among fever patients (Table 4).

#### Generic susceptibility indices

Generic susceptibility indices considered were sector of residency, been abroad within the last 3 years and wealth quintiles. The proportion of population who had been abroad within the last 3 years in the urban sector (4.5%, n = 99) was significantly higher than that of the rural (2.8%, n = 288) and estate sectors (0.2%, n = 2) (χ_4_^2^ = 66.103; p < 0.001) (Table 5).

**Table 5:** Distribution of population who had been abroad by sector of residence

Sector of residence	Been abroad within last 3 years			Total Number (%,95% CI)
	Number been abroad (%, 95% CI)	Number not been abroad (%, 95% CI)	Number don’t know (%,95% CI)	
Urban	99 (4.5, 3.7–5.5)	2082 (94.6,93.6–95.5)	20 (0.9, 0.6–1.4)	2201 (16.5, 15.8–17.1)
Rural	288 (2.8, 2.5–3.2)	9773 (95.5, 95.1–95.9)	170 (1.7, 1.4–1.9)	10,231 (76.6, 75.8–77.3)
Estate	2 (0.2, 0.03–0.8)	931 (99.8, 99.2–99.9)	0 (0.0)	933 (69.8, 65.5–74.3)
Total	389 (2.9, 2.6–3.2)	12,786 (95.7, 95.3–96.0)	190 (1.4, 1.2–1.6)	13,365 (100.0)

χ_4_^2^ = 66.103; p < 0.001

The proportion of the survey population who had been abroad within the last 3 years declined up to the 4th quintile; there was a slight rise in the 5th quintile (χ_8_2 = 60.985, P < 0.001) (Table 6).

**Table 6:** Association between having travelled abroad and wealth index

Wealth Quintiles	Been abroad within last 3 years			Total Number (%, 95% CI)
	Yes Number (%, 95% CI)	No Number (%, 95% CI)	Don’t know, Number (%, 95% CI)	
1st Quintile	109 (4.1,3.7–4.9)	2530 (94.5,93.6–95.3)	38 (1.4,1.0–1.9)	2677 (20.0,19.4–20.7)
2nd Quintile	89 (3.3, 2.7–4.1)	2583 (96.1, 95.3–96.8)	15 (0.6, 0.3–0.9)	2687 (20.1.19.4–20.8)
3rd Quintile	63 (2.4,1.8–3.0)	2559 (96.3, 95.5–97.0)	35 (1.3,0.9–1.8)	2657 (19.9,19.2–20.6)
4th Quintile	61 (2.3,1.8–2.9)	2578 (96.5, 95.7–97.1)	34 (1.3, 0.8–1.8)	2673 (20.0,19.3–20.7)
5th Quintile	67 (2.5, 1.9–3.2)	2536 (94.9,94.0–95.8)	68 (2.6, 2.0–3.2)	2671 (20.0,19.3–20.7)
Total	389 (2.9, 2.6–3.2)	12,786 (95.7, 95.3–96.0)	190 (1.4, 1.2–1.6)	13,365 (100.0)

Table 7 shows the summary of the binary logistic regression model using travel abroad as the dependent variable. The urban population was1.75 times more likely to travel abroad as compared to the rural/estate sector residents and males were 4.25 times more likely to travel than females. Persons in wealth quintile 5 were 1.5 times more likely to travel overseas as compared to persons from wealth quintiles 1 and 2 after controlling for age, sex and residence.

**Table 7:** Summary of binary logistic regression analysis using travel abroad as the dependent variable

	Regression Coefficient(B)	Standard error of regression coefficient	Wald statistic	Sig	Odds Ratio Exp(B)	95% CI of odds ratio	
						Lower	Upper
Gender^1^(male)	1.446	0.128	127.607	< 0.001	4.25	3.30	5.46
Area of residence^2^—urban	0.558	0.121	21.245	< 0.001	1.75	1.38	2.22
Age group^3^			127.280	< 0.001			
< 5 and > 65 years	− 0.665	0.147	20.583	< 0.001	0.51	0.39	0.69
6–35 years	− 1.458	0.131	122.951	< 0.001	0.23	0.18	0.30
Wealth Quintile ^4^			21.943	< 0.001			
Wealth Quintile 5	0.398	0.130	9.454	0.002	1.49	1.16	1.92
wealth Quintile 3 and 4	− 0.234	0.123	3.579	0.059	0.79	0.62	1.01
Constant	3.986	0.139	824.249				

^1^ Reference group is female

^2^ Reference group is rural and estate sector

^3^ Reference group is age group 35–65 years

^4^ Reference group is 1^st^ and 2^nd^ wealth quintiles

χ_8_^2^ = 60.985, P < 0.001

## Discussion

This study assessed the susceptibility to malaria at the national level in the prevention of re-establishment of malaria phase in Sri Lanka. In this study, as expected, persons’ resident in urban areas and in the upper wealth index had travelled overseas more than others. There was a decreasing trend in the proportion of persons travelling with increasing wealth quintile except in the lowest quintile which may be due to migration for labour. Not a single person had a malaria infection in the past three years. A previous study done during the pre-elimination phase in two formerly high endemic districts of Kurunegala and Anuradhapura reported that around 1.4% had a past history of malaria in the preceding 5 years of the survey [29]. A past history of malaria for three years was considered to rule out potential relapses that have been observed in imported cases after many months of their return to the country.

A number of epidemiologic studies support the link between malaria and migration. The prevalence of malaria is higher in communities with higher levels of immigration in the Amazon [30]. The migration of non-immune populations from Sri Lanka to malaria endemic countries influences the importation of malaria parasites into the country. In the past few years less than 100 imported cases have been reported annually. A reduction in the number of reported imported malaria cases during COVID-19 pandemic due to international travel restrictions in the recent past (year 2020/2021) has been observed.

Literature suggests that children under 5, persons more than 65 years and pregnant females are more susceptible to get malaria in a control setting. The relationship between malaria transmission and age is well documented by Molineaux, and by Snow and Marsh [31–34]. In high transmission settings, the incidence of malaria peaks in early childhood and then declines with increasing age with acquired immunity due to exposure to malaria infections. In moderate transmission settings, the age of peak transmission is a little later than childhood, while in low transmission settings, such as in Sri Lanka in the past, the risk of infection remains same across all ages [31]. In Sri Lanka, in the past, both children and adults developed acute clinical illness [35]. Lately, adults 15 and over is the main risk group for malaria [6]. However, Muhlberger et al., report that the risks of death and development of severe complications due to falciparum malaria increase with age in European patients who are non-immune to malaria [36]. Pregnant females are more prone to get infections as their immunological responses are lower due to the pregnancy [31, 36].

Age, sex and pregnancy status are considered as important factors in a control phase. In a prevention of re-establishment phase, all population groups will be susceptible due to waning immunity. The results of this study also suggest that, in a prevention of re-establishment phase, these indicators may not be useful indicators of susceptibility to malaria. These indicators were considered as important as if malaria is re-established these population groups will still be considered to be vulnerable. Based on the results, the most important factor for assessing susceptibility to malaria in the prevention of re-establishment phase is migration; recent travel overseas, wealth quintile and sector of residence are good indicators to monitor susceptibility to malaria.

Parasitaemia was considered as an indicator in the prevention of re-establishment phase specific for Sri Lanka due to large numbers of foreign workers in the country, re-settlement of refugees returning from India in the Northern Province and receptivity in certain areas of the country. As most non-migratory populations will be aparasitaemic in a non-transmission area, it is proposed that testing for malaria only be done among persons who have a history of travel within the last 3 years.

In the control phase, many factors have been associated with social vulnerability to malaria. In the highlands of East Africa, high levels of malaria vulnerability were found in the highlands where the immunity of the population was very low and in regions with lack of access to education and health services [17]. Lower values were found in regions with relatively low poverty, low population pressure and low conflict-density [17]. In Rwanda, the most influential susceptibility indicators were; population change (r = 0.729), average number of persons per bedroom (r = 0.531), number of households affected by droughts and famines (r = 0.591), area used for irrigation (r = 0.611), bed net ownership (r =  − 0.398) and poor housing wall materials (r = 0.378) [9]. The authors suggested that vulnerability assessments be done by combining environmental and social drivers to achieve an integrated and complete assessment and that spatial assessment be done without considering administrative boundaries.

As Sri Lanka was one of the first tropical countries in the recent past to eliminate malaria, there were no comparable studies to adapt methodological reproducibility in similar settings. Concept development depended heavily on the views of malaria experts in the country, studies done by experts in malaria control settings and disaster vulnerability studies. Other than vector and parasite factors, human factors in the form of social vulnerability are important to prevent re-establishment of malaria as highlighted in the conceptual framework.

This national survey to assess susceptibility to malaria during the prevention of re-establishment phase is the first study attempted in a country certified as malaria-free. The study did not specifically include or give priority for selection of specific migrant groups such as refugees, migrant workers residing in construction sites and factories. Refugee returnees from India in the Northern Province were included in the household survey as they were already re-settled and included in the sampling frame. It would have been better to include data on aspects such as areas of returning refugees, migrant labour and congregations of other high risk groups to assess generic susceptibility. Identifying these high risk populations is a challenge as there are illegal migrant workers in the country. Thus, there is an urgent need to identify migrant populations in the most vulnerable and receptive areas with potential for local transmission of malaria and to target interventions accordingly to prevent re-establishment of malaria in the country. One good example in the past was the asylum seekers residing in the Gampaha district, which was previously not endemic for malaria, where 17 imported malaria cases were reported in 2013; further local transmission was prevented through a high risk group screening programme [10].

The results from this study are important for public health policy and decision-makers in Sri Lanka. It also highlights the need for widening the scope of traditional malaria control, elimination and prevention of re-establishment programmes to include new aspects such as monitoring migration patterns and special areas where high risk groups, such as refugees and migrant foreign labour reside. This is a challenge and may require developing new procedures and task shifting of existing staff during the transition from a control to a prevention of re-establishment (PoR) phase. This special assessment provided timely evidence to plan and implement the prevention of re-establishment of malaria programme in Sri Lanka; the findings will improve the efficiency, effectiveness and sustainability of PoR interventions through advocacy, social mobilization and inter-sectoral collaboration to optimize the allocation of limited resources and health infrastructure at national level.

### Limitations

The data collected by the interviewer administered questionnaire in the survey were self-reported by the heads of households. Microscopy or RDT examination could not be done in some persons as they did not give consent for the procedure.

## Conclusions

Urban residents, upper socioeconomic class persons and male gender are more likely to travel overseas and bring the parasite into the country. The malaria screening and awareness programmes should be targeted to these populations. In addition, migration patterns and areas where known high risk groups reside should be monitored. This will require new procedures to be developed and task shifting of existing staff trained for a control phase operation to transit into a prevention of re-establishment phase when the programme is integrated with the general health services of the country eventually.

As susceptibility to malaria is a dynamic phenomenon, susceptibility to malaria should be assessed every 3–5 years. As baseline data is now available, the Regional Malaria Officers can update the required information. Susceptibility assessments should combine with resilience and receptivity to assess social vulnerability and risk of malaria during prevention of re-establishment of malaria.

## Data Availability

All data generated or analysed during this study are available on request.

## Abbreviations

A/L: Advanced level
AMC: Anti Malaria Campaign
DHS: Demographic Health Survey
GCE: General Certificate of Education
GPS: Global Positioning System
IOM: International Organization for Migration
MOH: Medical Officer of Health
MOVE: Methods for the Improvement of Vulnerability Assessment in Europe
O/L: Ordinary Level
PCA: Principal components analysis
PCR: Polymerase Chain reaction
PHM: Public Health Midwife
PoR: Prevention of Re-establishment
RDT: Rapid Diagnostic test
SES: Socio economic status
SLR: Sri Lanka Rupees
SPSS: Statistical Package for Social Sciences
WHO: World Health Organization
